# Effects of exercise and diet-induced weight loss on markers of inflammation I: impact on body composition and markers of health and fitness

**DOI:** 10.1186/1550-2783-10-S1-P15

**Published:** 2013-12-06

**Authors:** K Levers, S Simbo, B Lockard, C Baetge, E Galvan, M Byrd, YP Jung, A Jagim, JM Oliver, M Koozehchian, R Dalton, D Khanna, B Sanchez, JY Kresta, K Horrell, T Leopold, M Cho, S Springer, A Rivera, C Cerda, C Rasmussen, R Kreider

**Affiliations:** 1 Exercise & Sport Nutrition Lab, Texas A&M University, College Station, TX 77843, USA

## Background

The purpose of this study was to determine the effects of participating in a resistance-exercise based circuit training program while adhering to a higher protein diet designed to preserve fat free mass (FFM) during weight loss on body composition and markers of health. Then, in a companion paper, determine if exercise and diet-induced weight loss affect markers of inflammation.

## Methods

48 sedentary women (48.2±10.5 yr, 45.9±4.4% body fat, 35.6±5.6kg/m^2^) were randomized to participate in the Curves® weight loss and exercise program (EX, n=28) or control group (C, n=20) for 12-wks. Participants followed an energy-restricted diet (1,200 kcal/d for 1-week and 1,500 kcal/d for 11 weeks; 30% CHO, 45% P, and 25% F) while participating in a circuit resistance-training (4 d/wk) program. On one of the four exercise days, Zumba® dance was interspersed with the circuit resistance stations, wherein participants completed 60 seconds of resistance exercise followed by 60 seconds of dance. On the other three days of the 4 d/wk program, the workout included 30 seconds of resistance-exercise interspersed with 30 seconds of continuous movement (calisthenics, dance, etc.). DEXA body composition and fasting blood samples were obtained at 0 and 12-wks and analyzed by MANOVA. Data are presented as changes from baseline after 12-wks for the EX and C groups.

## Results

Overall MANOVA analysis revealed a significant group x time effect (p=0.004) for body composition measures. Univariate analysis revealed that participants in the EX group experienced greater changes in body weight (EX -4.0±4.4 kg; C 0.1±3.0 kg, p=0.001), fat mass (EX -3.8±4.0 kg; C -0.03±2.0 kg, p<0.001), and percent body fat (EX -2.7±3.4%; C -0.1±1.7%, p=0.002). No differences among groups were observed in FFM (EX -0.2±2.0 kg; C 0.1±2.3 kg, p=0.59). Overall MANOVA analysis revealed a non-significant group x time effect (p=0.21) for blood markers. Although positive trends were observed, univariate analysis revealed no significant differences among groups for triglycerides (EX -6.7±26.4%; C 0.1±24.4%, p=0.37), total cholesterol (EX -3.6±10.0%; C -2.2±10.7%, p=0.65), high density lipoprotein cholesterol (EX 2.5±15.1%; C -5.0±10.5%, p=0.06); low-density lipoprotein cholesterol (EX -4.7±11.5%; C -4.0±16.8%, p=0.86) or blood glucose (EX -0.6±14.5%; C -1.3±8.4%, p=0.85). Overall MANOVA analysis revealed a significant group x time effect (p=0.003) for measures of fitness. Univariate analysis revealed that participants in EX group experienced greater changes in peak oxygen uptake (EX 13.6±17.0%; C -2.2±10.3%, p=0.001) and upper body 1-RM strength (EX 8.7±12.5%; C -1.2±13.9%, p=0.016) while no differences were observed among groups in changes in lower body 1-RM strength (EX 15.0±21.9%; C 13.8±23.7%, p=0.86).

## Conclusion

Results indicate that 12-wks of participation in the exercise and diet-induced weight loss program involving a structured meal plan and a supervised exercise program promoted weight loss, improvements in body composition, and improvements in some markers of health and fitness. Theoretically, if obesity is associated with inflammation, effective weight loss may lessen levels of inflammation.

